# Advanced Squamous Cell Carcinoma in the Paratracheal Region: Navigating Diagnosis and Comprehensive Treatment Challenges

**DOI:** 10.7759/cureus.72612

**Published:** 2024-10-29

**Authors:** Nathaniel Grabill, Mena Louis, Deepak Vivekanandan, Jerrell Fang, J Clifton Hastings

**Affiliations:** 1 General Surgery, Northeast Georgia Medical Center Gainesville, Gainesville, USA; 2 Cardiothoracic Surgery, Northeast Georgia Medical Center Gainesville, Gainesville, USA

**Keywords:** airway management, chemotherapy, dysphagia, paratracheal mass, squamous cell carcinoma

## Abstract

This case of a paratracheal mass emphasizes the importance of early detection and flexibility in the treatment planning for advanced squamous cell carcinoma, especially when logistical challenges impact access to care. A 69-year-old woman presented with a four-month history of progressive dysphagia, significant weight loss, and the recent onset of stridor, suggesting potential airway obstruction. Imaging studies revealed a large heterogeneous mass in the superior mediastinum, extending from the base of the neck into the thoracic inlet. The mass measured approximately 6.9 cm × 3.4 cm, involving the trachea and upper esophagus, causing significant compression and deviation. Additional findings included small hypermetabolic lymph nodes in the mediastinum. The patient underwent bronchoscopy and endoscopy, which revealed narrowing but no intraluminal lesions, indicating external compression by the mass. The pathological examination confirmed well-differentiated squamous cell carcinoma, characterized by keratinization and positive p40 immunostaining, with negative p16 immunostaining, indicating a non-HPV-related etiology. An urgent tracheostomy was performed to secure the airway, which the patient tolerated well. Following this, inpatient chemotherapy with a regimen of Taxotere, Cisplatin, and 5-fluorouracil (TPF) was initiated to manage the tumor and prevent further complications. This case required a multidisciplinary approach to address the patient's complex clinical presentation, including surgical, oncological, and supportive care. As confirmed by imaging, the lack of distant metastasis suggests a potentially better prognosis if effective local control can be achieved. The integrated care provided was essential in managing the patient's immediate and long-term health needs.

## Introduction

Paratracheal masses are abnormal growths adjacent to the trachea, often presenting a diagnostic and therapeutic challenge due to their proximity to vital structures such as the airway, esophagus, and major blood vessels [1,2]. These masses can be benign or malignant and may arise from various tissues, including lymph nodes, thyroid, or metastatic sites [1]. The clinical presentation of a paratracheal mass often depends on its size and anatomical involvement, with symptoms potentially including respiratory distress, dysphagia, and even vascular compression [3]. The management of paratracheal masses requires a careful assessment to determine the nature of the mass and the best therapeutic approach, which may involve surgery, radiation, chemotherapy, or a combination of these treatments [4,5].

Squamous cell carcinoma of the esophagus is a type of cancer that arises from the squamous cells lining the esophagus [6]. It is one of the most common types of esophageal cancer and is strongly associated with risk factors such as tobacco use and alcohol consumption [7]. This malignancy often presents late due to initially subtle symptoms, including difficulty swallowing (dysphagia), which can progressively worsen as the tumor grows [8]. Early detection and intervention are crucial for improving prognosis, as squamous cell carcinoma of the esophagus tends to invade nearby structures and spread to regional lymph nodes [9]. Treatment options typically include surgery, chemotherapy, and radiation therapy, depending on the stage of the disease and the patient's overall health [10].

Compression and deviation of the trachea and esophagus due to a mass can lead to significant clinical symptoms and complications [11]. Such masses can cause mechanical obstruction, leading to symptoms such as stridor, coughing, difficulty breathing, and dysphagia [10]. In severe cases, tracheal compression can result in airway compromise, necessitating urgent interventions such as tracheostomy [12]. Dysphagia, or difficulty swallowing, is particularly concerning in cancer patients, as it can lead to malnutrition, dehydration, and a decline in overall health status [13]. The management of these complications often requires a multidisciplinary approach, including nutritional support, prevention of aspiration, surgical interventions to relieve obstruction, and targeted cancer therapies to reduce tumor burden [14].

## Case presentation

A 69-year-old woman presented to the hospital with a four-month history of progressive dysphagia, significant weight loss, and the recent onset of audible stridor. The patient's symptoms began with mild difficulty swallowing, which worsened over time, restricting her to only small bites of food and leading to a 22-pound weight loss. Upon admission, the presence of stridor raised concerns about potential airway obstruction.

**Figure 1:**
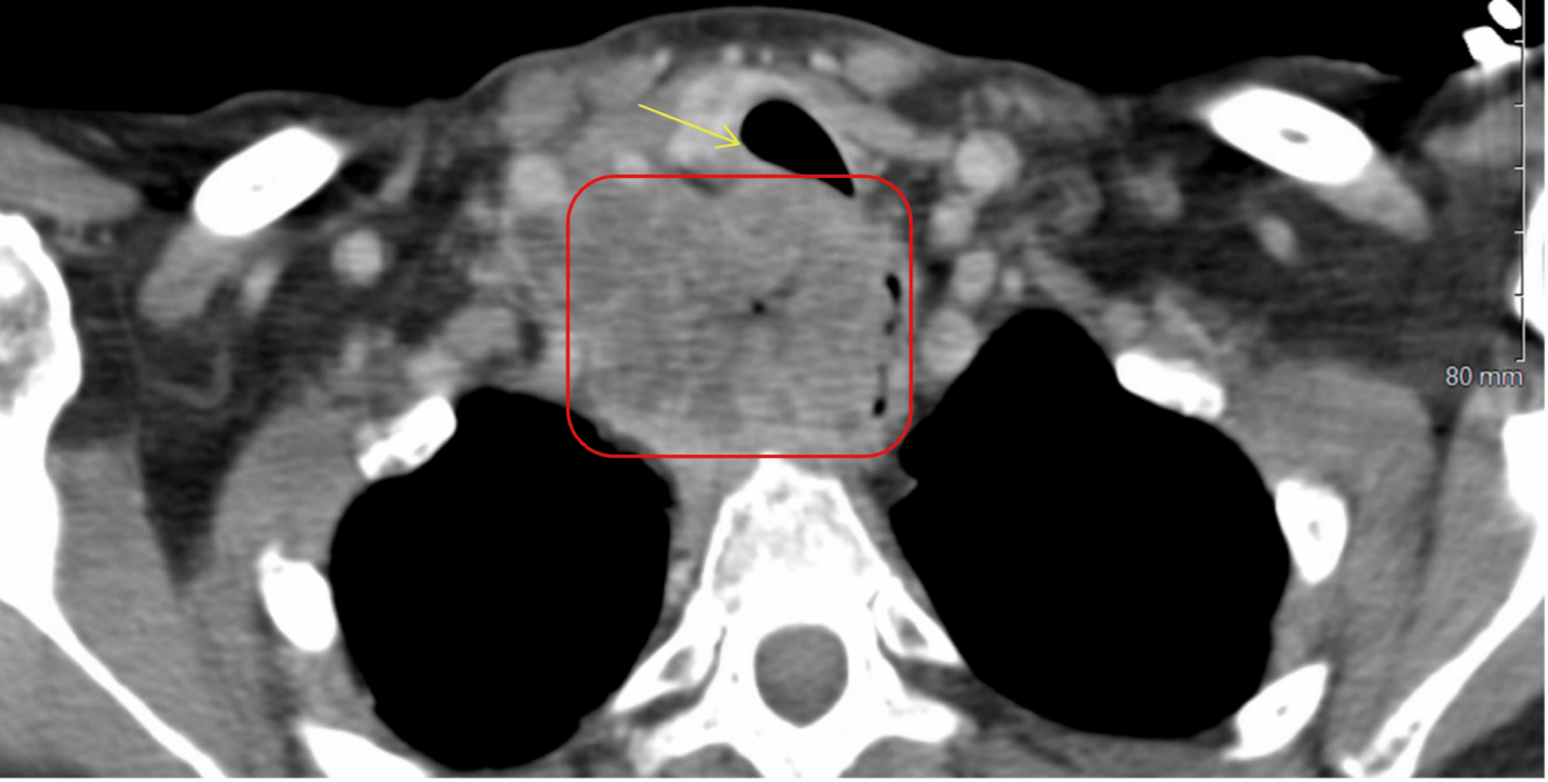
The CTA pulmonary (axial view) shows a large right paratracheal mass (red box), with an impression on the trachea and esophagus, with a deviation to the left (yellow arrow). There is a tracheal invasion within the posterior midsegment with approximately 70% stenosis of the trachea at this point. CTA: computed tomographic angiography

**Figure 2:**
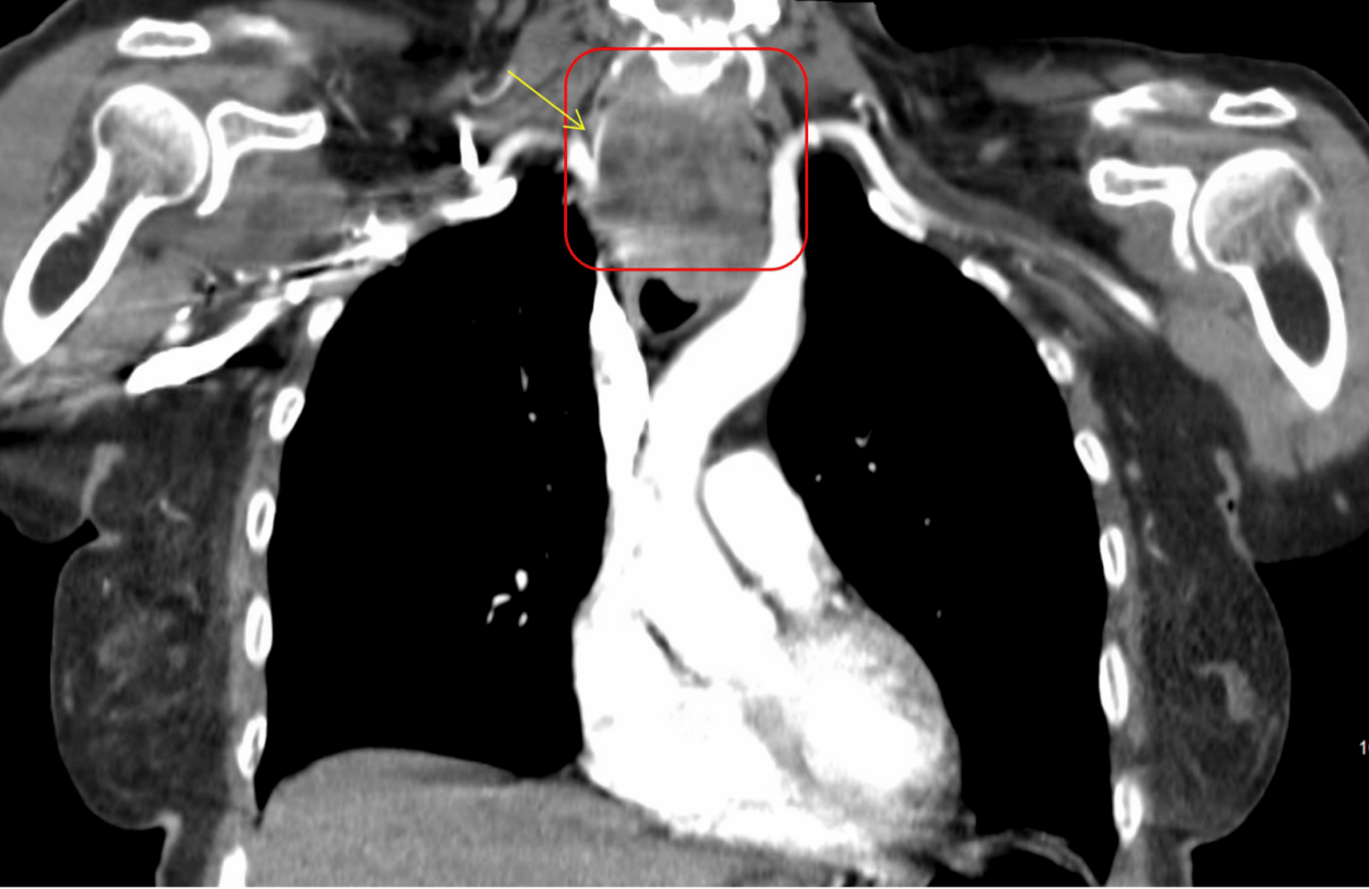
The CTA pulmonary (coronal view) shows a large right paratracheal mass (red box). The mass demonstrates a superior lateral extension into the right supraclavicular fossa, with near complete encasement of the proximal right common carotid artery (yellow arrow). CTA: computed tomographic angiography

CT imaging studies identified a large heterogeneous mass in the superior mediastinum extending from the base of the neck into the thoracic inlet. The mass measured approximately 6.9 cm anteroposteriorly and 3.4 cm transversely (Figures 1, 2), with intense hypermetabolic activity (maximum standardized uptake value (SUVmax) of 14.3). This mass involved the trachea and upper third of the esophagus, causing significant compression and deviation of these structures. Importantly, there was no evidence of distant metastatic disease, as the liver, adrenal glands, lungs, and bones showed no abnormal uptake.

The patient had a notable history of smoking one to two cigarettes per day for 15 years, having quit 10 years prior. There was no significant family history of cancer. Bronchoscopy performed during evaluation did not reveal any intraluminal lesions in the trachea, and endoscopy showed esophageal narrowing without mucosal abnormalities (no images available), suggesting that the symptoms were due to external compression by the mass.

Given the risk of airway compromise indicated by the stridor and imaging findings, an urgent tracheostomy was performed. This procedure was not emergent, allowing time for thorough evaluation and preparation, ensuring the patient's stability during the procedure. The patient tolerated the tracheostomy well, which secured her airway and alleviated the immediate risk of respiratory distress.

**Figure 3:**
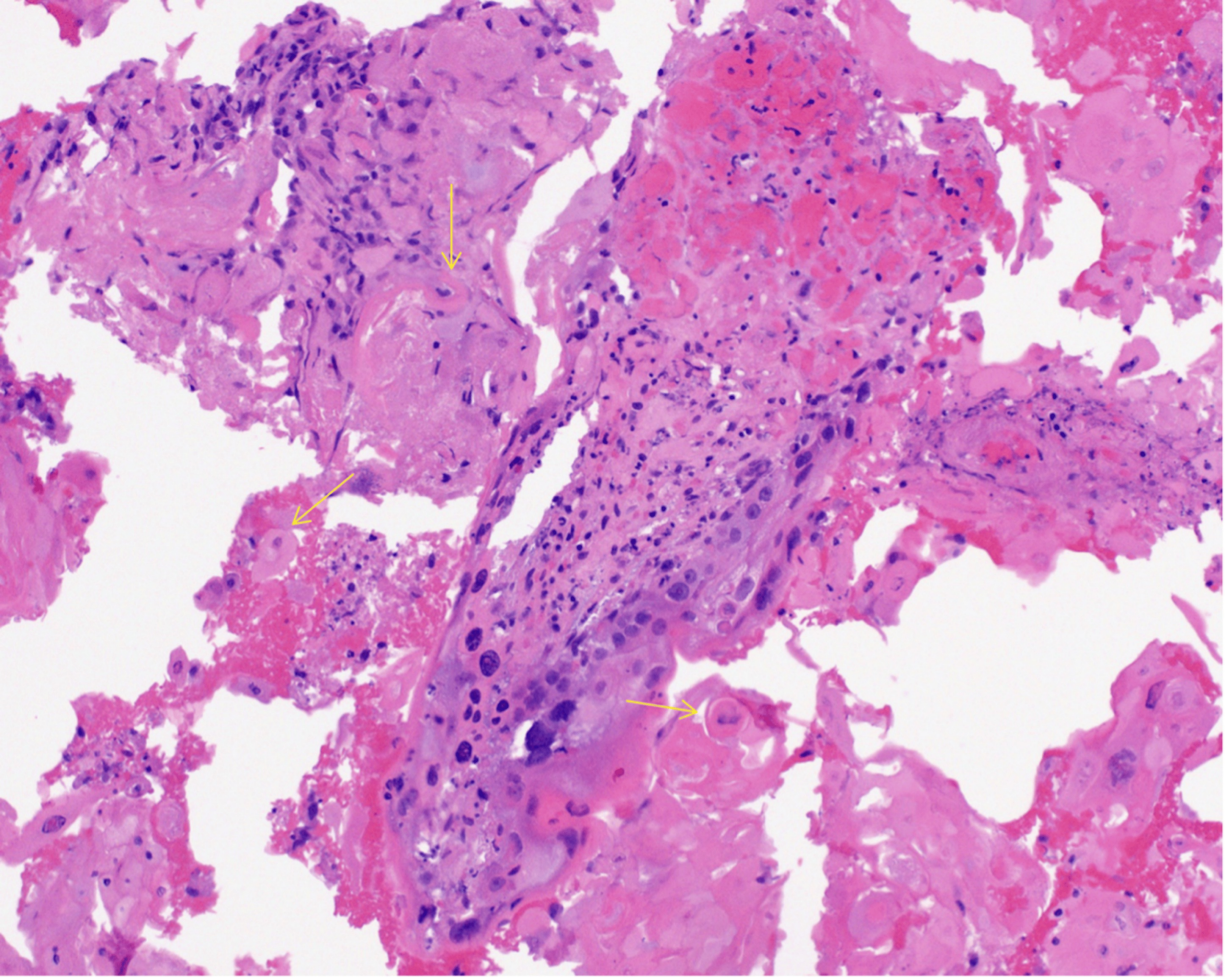
This H&E-stained section at 20× magnification reveals squamous cell carcinoma with keratin pearls (yellow arrows). H&E: hematoxylin and eosin

**Figure 4:**
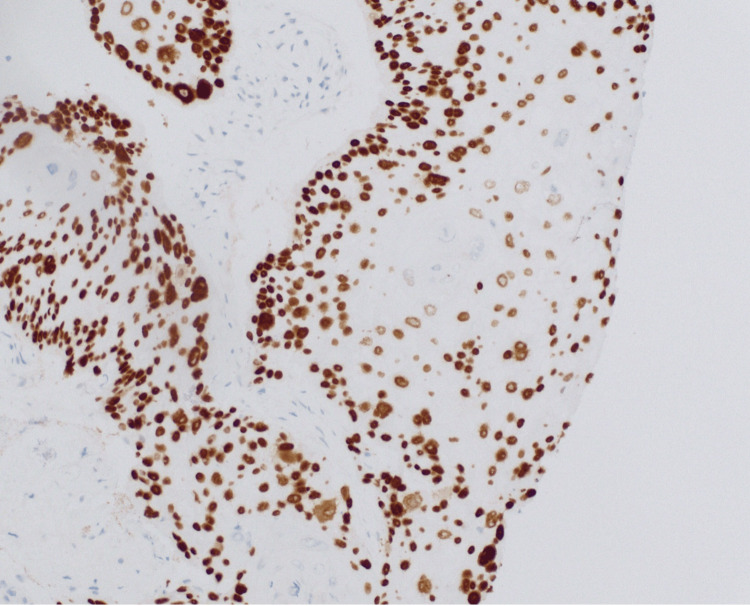
This slide at 20× magnification with positive p40 immunostaining is supportive of squamous cell carcinoma diagnosis.

The pathological examination of fine-needle aspiration biopsies confirmed the presence of well-differentiated squamous cell carcinoma, with characteristic keratinization (Figure 3) and positive p40 immunostaining (Figure 4), confirming the squamous cell origin. The p16 immunostain was negative, indicating that the carcinoma was not associated with human papillomavirus (HPV) infection.

The pathology findings from the fine-needle aspiration biopsies of the right neck mass confirmed the presence of well-differentiated squamous cell carcinoma, characterized by keratinization (Figure 3) and positive p40 immunostaining (Figure 4), which is indicative of squamous cell lineage. The p16 immunostain was essentially negative, suggesting that the tumor may not be associated with HPV, which can be a significant factor in the etiology and prognosis of head and neck cancers. The well-differentiated nature of the carcinoma indicates a more organized cellular structure, which may influence treatment decisions and prognosis. Clinical correlation was recommended to identify the primary site, as squamous cell carcinoma can arise in various head and neck regions, making accurate localization essential for targeted therapy.

The patient was started on an inpatient chemotherapy regimen, including Taxotere, Cisplatin, and 5-fluorouracil (TPF), to manage the primary tumor and control symptoms. This approach was necessary due to insurance-related delays in starting radiation therapy. The chemotherapy was monitored closely to detect any potential adverse reactions or worsening symptoms.

The comprehensive management of this case involved a multidisciplinary team approach, addressing the immediate airway and nutritional needs while planning for ongoing oncological treatment. The absence of distant metastasis provided a potentially better prognosis, contingent on effective local disease control. The patient's significant dysphagia and weight loss necessitated the placement of a percutaneous endoscopic gastrostomy (PEG) tube for nutritional support. This intervention was crucial in ensuring that the patient received adequate nutrition, as her ability to consume food orally was severely compromised by the tumor's compression of the esophagus.

Despite extensive imaging and diagnostic efforts, including bronchoscopy and endoscopy (no images available), the primary tumor site was not definitively identified. The mass was located in the superior mediastinum, involving both the trachea and upper esophagus, but neither the trachea nor the esophagus showed direct mucosal involvement, which made the exact origin of the tumor unclear. Given that the mass abutted the trachea and the esophagus and was causing a significant external mass effect, the multidisciplinary team did not think it was immediately resectable. The team felt the best approach was to start radiation therapy with the hope of shrinking the mass to make it potentially resectable in the future.

Inpatient chemotherapy was initiated using a TPF regimen (Taxotere, Cisplatin, and 5-fluorouracil) to address the tumor's aggressive nature and manage symptoms. This decision was influenced by insurance limitations that delayed immediate access to radiation therapy. The treatment aimed to reduce the tumor burden and prevent further complications, with plans for outpatient radiation therapy and continued chemotherapy. The patient's condition required ongoing multidisciplinary care, including oncological, surgical, and supportive measures, to manage the complex clinical presentation and ensure comprehensive care. The patient was subsequently discharged home. The patient is currently on weekly Cisplatin and radiation.

## Discussion

The presentation of a 69-year-old woman with audible stridor during admission indicated a potential airway obstruction, necessitating immediate medical attention. Imaging studies revealed a large hypermetabolic mass in the superior mediastinum, extending from the base of the neck into the thoracic inlet, involving both the trachea and upper esophagus. This mass caused significant compression and deviation of the trachea, along with small hypermetabolic lymph nodes noted in the mediastinum.

A bronchoscopy conducted during the tracheostomy procedure did not reveal any intraluminal lesions in the trachea, while an endoscopy showed no mucosal lesions, only narrowing due to external compression. The patient's history of smoking and lack of a family history of cancer were notable risk factors for squamous cell carcinoma. However, the tumor's origin, whether tracheal or esophageal, was not determined.

An urgent tracheostomy was performed one day after admission, allowing for thorough evaluation and preparation, thus avoiding an emergent procedure. This approach proved beneficial, stabilizing the patient's airway and preventing respiratory distress. The patient tolerated the tracheostomy well, indicating the effectiveness of the planned intervention.

The literature has also discussed the use of endobronchial stents to relieve stridor due to subglottic stenosis of malignant etiology [15-17]. However, endobronchial stents have limitations, including preventing the delivery of appropriate radiation doses due to interaction with localization imaging and the inability to ascertain response to treatment due to interaction with imaging [18,19].

Pathological analysis confirmed well-differentiated squamous cell carcinoma with positive p40 immunostaining and negative p16 immunostaining, suggesting a non-HPV-related origin. This information was crucial for guiding treatment, as HPV status significantly influences the management and prognosis of squamous cell carcinomas.

The standard of care for management of squamous cell carcinoma (esophageal origin) includes 50.4 Gy of radiation along with weekly Cisplatin and 5-fluorouracil, with additional fractions up to 66 Gy depending on response [6,8]. However, patients with primary head and neck squamous cell carcinoma have been managed with a median dose of 70 Gy radiation with simultaneous chemotherapy, most commonly Mitomycin C and 5-fluorouracil, in concordance with the ARO 95-06 trial.

To manage the significant tumor burden in this patient and preempt potential complications, the patient was started on an inpatient chemotherapy regimen with TPF. This treatment was necessary due to insurance-related delays in accessing radiation therapy, allowing for close monitoring of any adverse reactions or worsening symptoms. The inpatient setting provided the capability to respond promptly to any complications from the treatment.

The comprehensive management of this patient's superior mediastinal squamous cell carcinoma involved a coordinated, multidisciplinary approach. The lack of distant metastasis, as confirmed by PET scans, provided a more optimistic outlook, provided that effective local control of the disease could be achieved.

## Conclusions

In cases involving superior mediastinal squamous cell carcinoma, early detection and comprehensive management are critical, especially when vital structures such as the trachea and esophagus are involved. The presence of symptoms such as progressive dysphagia, significant weight loss, and stridor necessitates prompt evaluation and intervention. A multidisciplinary approach, including airway management through tracheostomy and chemotherapy, is essential in addressing both immediate and long-term care needs. Challenges such as insurance-related delays in treatment highlight the need for adaptable treatment plans. The absence of distant metastasis can be a positive prognostic factor if effective local control of the disease is achieved.
